# Supplementary material for: Modeling glioblastoma heterogeneity as a dynamic network of cell states
Source: Mol Syst Biol. 2021 Sep 16;17(9):e10105. doi: 10.15252/msb.202010105 (PMC8444284; doi:10.15252/msb.202010105)
Supplement: Supplementary file 6 — Source Data for Figure 5 [file MSB-17-e10105-s004.zip › Figure5A_sourcedata/GSEA_3017/nosigdb_stateD.GseaPreranked.1622795010397/RICHARDS_NOWAKOWSKI_SCIENCE_2017_U4_UPREG.html]

Details for gene set RICHARDS\_NOWAKOWSKI\_SCIENCE\_2017\_U4\_UPREG[GSEA]

|  || Dataset | state23017 |
| Phenotype | NoPhenotypeAvailable |
| Upregulated in class | na\_pos |
| GeneSet | RICHARDS\_NOWAKOWSKI\_SCIENCE\_2017\_U4\_UPREG |
| Enrichment Score (ES) | 0.04875734 |
| Normalized Enrichment Score (NES) | 0.5465486 |
| Nominal p-value | 1.0 |
| FDR q-value | 0.98582786 |
| FWER p-Value | 1.0 |
Table: GSEA Results Summary

  

Fig 1: Enrichment plot: RICHARDS\_NOWAKOWSKI\_SCIENCE\_2017\_U4\_UPREG      
 Profile of the Running ES Score & Positions of GeneSet Members on the Rank Ordered List

  

| PROBE | GENE SYMBOL | GENE\_TITLE | RANK IN GENE LIST | RANK METRIC SCORE | RUNNING ES | CORE ENRICHMENT || 1 | HAS2 |  |  | 4 | 0.596 | 0.0053 | Yes |
| 2 | PDGFRA |  |  | 8 | 0.551 | 0.0108 | Yes |
| 3 | OLIG1 |  |  | 10 | 0.517 | 0.0176 | Yes |
| 4 | DSEL |  |  | 16 | 0.472 | 0.0202 | Yes |
| 5 | PTMS |  |  | 20 | 0.452 | 0.0242 | Yes |
| 6 | MIR4458HG |  |  | 22 | 0.446 | 0.0299 | Yes |
| 7 | VMP1 |  |  | 26 | 0.414 | 0.0334 | Yes |
| 8 | HSP90AB1 |  |  | 32 | 0.393 | 0.0348 | Yes |
| 9 | PURB |  |  | 41 | 0.370 | 0.0332 | Yes |
| 10 | BTG1 |  |  | 50 | 0.350 | 0.0313 | Yes |
| 11 | HSPB1 |  |  | 52 | 0.347 | 0.0356 | Yes |
| 12 | TGIF1 |  |  | 55 | 0.343 | 0.0389 | Yes |
| 13 | RPL10A |  |  | 65 | 0.327 | 0.0358 | Yes |
| 14 | SRRT |  |  | 74 | 0.314 | 0.0333 | Yes |
| 15 | VIM |  |  | 75 | 0.314 | 0.0380 | Yes |
| 16 | TRIO |  |  | 76 | 0.314 | 0.0426 | Yes |
| 17 | CALM3 |  |  | 82 | 0.308 | 0.0428 | Yes |
| 18 | EIF5A |  |  | 90 | 0.301 | 0.0411 | Yes |
| 19 | GDF11 |  |  | 97 | 0.294 | 0.0401 | Yes |
| 20 | OLIG2 |  |  | 98 | 0.293 | 0.0444 | Yes |
| 21 | RPL22 |  |  | 99 | 0.292 | 0.0488 | Yes |
| 22 | RPS11 |  |  | 113 | 0.279 | 0.0414 | No |
| 23 | CHD7 |  |  | 116 | 0.278 | 0.0438 | No |
| 24 | GTPBP4 |  |  | 122 | 0.275 | 0.0434 | No |
| 25 | RPL6 |  |  | 134 | 0.267 | 0.0376 | No |
| 26 | PPP1R15A |  |  | 149 | 0.259 | 0.0291 | No |
| 27 | LRRN1 |  |  | 151 | 0.258 | 0.0320 | No |
| 28 | RPS16 |  |  | 158 | 0.251 | 0.0304 | No |
| 29 | PPM1D |  |  | 162 | -0.251 | 0.0315 | No |
| 30 | PPP1R12B |  |  | 174 | -0.252 | 0.0255 | No |
| 31 | RHBDD2 |  |  | 192 | -0.253 | 0.0142 | No |
| 32 | METTL4 |  |  | 195 | -0.253 | 0.0162 | No |
| 33 | TICRR |  |  | 199 | -0.253 | 0.0173 | No |
| 34 | SUCLG1 |  |  | 200 | -0.253 | 0.0210 | No |
| 35 | GPAA1 |  |  | 204 | -0.254 | 0.0221 | No |
| 36 | SREK1 |  |  | 212 | -0.255 | 0.0197 | No |
| 37 | NAP1L4 |  |  | 215 | -0.255 | 0.0217 | No |
| 38 | RFTN2 |  |  | 219 | -0.255 | 0.0228 | No |
| 39 | CALCOCO2 |  |  | 227 | -0.256 | 0.0204 | No |
| 40 | TPM2 |  |  | 229 | -0.257 | 0.0234 | No |
| 41 | BTBD1 |  |  | 232 | -0.257 | 0.0254 | No |
| 42 | AARS |  |  | 234 | -0.257 | 0.0283 | No |
| 43 | BTBD6 |  |  | 237 | -0.257 | 0.0304 | No |
| 44 | PSAT1 |  |  | 241 | -0.258 | 0.0316 | No |
| 45 | CRYBG3 |  |  | 244 | -0.259 | 0.0336 | No |
| 46 | KAT7 |  |  | 248 | -0.259 | 0.0348 | No |
| 47 | AKIRIN2 |  |  | 269 | -0.261 | 0.0210 | No |
| 48 | PRKD1 |  |  | 273 | -0.261 | 0.0222 | No |
| 49 | COLGALT2 |  |  | 275 | -0.261 | 0.0252 | No |
| 50 | PIK3R3 |  |  | 277 | -0.261 | 0.0282 | No |
| 51 | RFX2 |  |  | 280 | -0.262 | 0.0303 | No |
| 52 | MTHFD1 |  |  | 281 | -0.262 | 0.0341 | No |
| 53 | FGF2 |  |  | 287 | -0.263 | 0.0336 | No |
| 54 | SNX13 |  |  | 301 | -0.264 | 0.0260 | No |
| 55 | RMDN3 |  |  | 302 | -0.264 | 0.0299 | No |
| 56 | LGR4 |  |  | 305 | -0.264 | 0.0321 | No |
| 57 | P4HTM |  |  | 313 | -0.265 | 0.0298 | No |
| 58 | MSI1 |  |  | 332 | -0.267 | 0.0178 | No |
| 59 | CYFIP2 |  |  | 334 | -0.267 | 0.0209 | No |
| 60 | ACAP2 |  |  | 367 | -0.270 | -0.0034 | No |
| 61 | C2orf80 |  |  | 374 | -0.271 | -0.0047 | No |
| 62 | RPN2 |  |  | 376 | -0.271 | -0.0016 | No |
| 63 | COPRS |  |  | 380 | -0.271 | -0.0002 | No |
| 64 | ST5 |  |  | 392 | -0.272 | -0.0059 | No |
| 65 | MED27 |  |  | 397 | -0.273 | -0.0054 | No |
| 66 | NPDC1 |  |  | 402 | -0.274 | -0.0049 | No |
| 67 | FH |  |  | 408 | -0.274 | -0.0053 | No |
| 68 | INTS10 |  |  | 409 | -0.274 | -0.0012 | No |
| 69 | GLIPR2 |  |  | 411 | -0.275 | 0.0020 | No |
| 70 | ERICH3 |  |  | 429 | -0.277 | -0.0090 | No |
| 71 | TOPBP1 |  |  | 439 | -0.278 | -0.0128 | No |
| 72 | VOPP1 |  |  | 441 | -0.279 | -0.0096 | No |
| 73 | TUBGCP5 |  |  | 443 | -0.279 | -0.0063 | No |
| 74 | EP400 |  |  | 451 | -0.279 | -0.0084 | No |
| 75 | GJA1 |  |  | 453 | -0.280 | -0.0051 | No |
| 76 | BRD2 |  |  | 461 | -0.281 | -0.0071 | No |
| 77 | LCORL |  |  | 464 | -0.281 | -0.0047 | No |
| 78 | IFRD1 |  |  | 469 | -0.282 | -0.0041 | No |
| 79 | PCNT |  |  | 485 | -0.283 | -0.0132 | No |
| 80 | ZGRF1 |  |  | 499 | -0.285 | -0.0205 | No |
| 81 | ERC1 |  |  | 507 | -0.286 | -0.0224 | No |
| 82 | SLC3A2 |  |  | 517 | -0.287 | -0.0261 | No |
| 83 | LRP1B |  |  | 519 | -0.287 | -0.0228 | No |
| 84 | LRRFIP2 |  |  | 520 | -0.287 | -0.0185 | No |
| 85 | RHNO1 |  |  | 523 | -0.288 | -0.0160 | No |
| 86 | MAD1L1 |  |  | 525 | -0.288 | -0.0126 | No |
| 87 | LUZP2 |  |  | 535 | -0.289 | -0.0163 | No |
| 88 | TAOK3 |  |  | 536 | -0.289 | -0.0120 | No |
| 89 | TRRAP |  |  | 537 | -0.289 | -0.0077 | No |
| 90 | GAB1 |  |  | 542 | -0.290 | -0.0070 | No |
| 91 | ARFGAP3 |  |  | 545 | -0.290 | -0.0044 | No |
| 92 | PHLPP1 |  |  | 549 | -0.291 | -0.0028 | No |
| 93 | DYRK1A |  |  | 551 | -0.291 | 0.0006 | No |
| 94 | WDR76 |  |  | 557 | -0.292 | 0.0005 | No |
| 95 | NUP85 |  |  | 562 | -0.292 | 0.0013 | No |
| 96 | LZTS1 |  |  | 564 | -0.293 | 0.0048 | No |
| 97 | SIVA1 |  |  | 567 | -0.293 | 0.0074 | No |
| 98 | EMC7 |  |  | 576 | -0.295 | 0.0047 | No |
| 99 | MPRIP |  |  | 577 | -0.296 | 0.0090 | No |
| 100 | TANC2 |  |  | 583 | -0.296 | 0.0090 | No |
| 101 | MLF1 |  |  | 585 | -0.297 | 0.0125 | No |
| 102 | ACTN4 |  |  | 604 | -0.301 | 0.0011 | No |
| 103 | CCDC93 |  |  | 610 | -0.303 | 0.0011 | No |
| 104 | NCAPH2 |  |  | 616 | -0.303 | 0.0012 | No |
| 105 | PAPSS1 |  |  | 621 | -0.304 | 0.0022 | No |
| 106 | MCM3 |  |  | 625 | -0.305 | 0.0040 | No |
| 107 | ZHX3 |  |  | 642 | -0.309 | -0.0056 | No |
| 108 | CRY1 |  |  | 654 | -0.311 | -0.0107 | No |
| 109 | MEG8 |  |  | 658 | -0.311 | -0.0087 | No |
| 110 | FZD5 |  |  | 661 | -0.312 | -0.0059 | No |
| 111 | PHGDH |  |  | 662 | -0.312 | -0.0012 | No |
| 112 | RAB31 |  |  | 667 | -0.313 | -0.0001 | No |
| 113 | PPP1R9A |  |  | 675 | -0.315 | -0.0017 | No |
| 114 | TUBB2A |  |  | 677 | -0.315 | 0.0021 | No |
| 115 | TRIM37 |  |  | 689 | -0.317 | -0.0029 | No |
| 116 | SHROOM3 |  |  | 693 | -0.318 | -0.0009 | No |
| 117 | SNTG1 |  |  | 697 | -0.319 | 0.0012 | No |
| 118 | NCOA1 |  |  | 700 | -0.319 | 0.0042 | No |
| 119 | S100B |  |  | 705 | -0.320 | 0.0054 | No |
| 120 | RAB10 |  |  | 708 | -0.321 | 0.0084 | No |
| 121 | NOS1AP |  |  | 727 | -0.324 | -0.0028 | No |
| 122 | ACTN1 |  |  | 729 | -0.324 | 0.0011 | No |
| 123 | PCDH19 |  |  | 749 | -0.331 | -0.0108 | No |
| 124 | EXTL3 |  |  | 753 | -0.332 | -0.0085 | No |
| 125 | HADH |  |  | 754 | -0.333 | -0.0036 | No |
| 126 | LIFR |  |  | 755 | -0.333 | 0.0014 | No |
| 127 | OSBPL6 |  |  | 758 | -0.333 | 0.0045 | No |
| 128 | ASRGL1 |  |  | 760 | -0.333 | 0.0086 | No |
| 129 | PRIM2 |  |  | 764 | -0.336 | 0.0109 | No |
| 130 | CTDSPL |  |  | 768 | -0.336 | 0.0132 | No |
| 131 | GCLM |  |  | 770 | -0.337 | 0.0173 | No |
| 132 | GOLGB1 |  |  | 775 | -0.338 | 0.0188 | No |
| 133 | BRD8 |  |  | 778 | -0.338 | 0.0221 | No |
| 134 | AHI1 |  |  | 806 | -0.347 | 0.0033 | No |
| 135 | GNB5 |  |  | 827 | -0.351 | -0.0092 | No |
| 136 | IFI27L1 |  |  | 829 | -0.352 | -0.0049 | No |
| 137 | KIF1A |  |  | 835 | -0.354 | -0.0041 | No |
| 138 | RRM2B |  |  | 839 | -0.355 | -0.0014 | No |
| 139 | TIPARP |  |  | 841 | -0.355 | 0.0029 | No |
| 140 | EPS8 |  |  | 843 | -0.356 | 0.0073 | No |
| 141 | INSR |  |  | 846 | -0.356 | 0.0108 | No |
| 142 | ABHD3 |  |  | 848 | -0.357 | 0.0152 | No |
| 143 | ZNF521 |  |  | 851 | -0.358 | 0.0188 | No |
| 144 | CLSTN1 |  |  | 855 | -0.359 | 0.0214 | No |
| 145 | DTNA |  |  | 857 | -0.359 | 0.0259 | No |
| 146 | STK39 |  |  | 858 | -0.359 | 0.0312 | No |
| 147 | MEST |  |  | 864 | -0.360 | 0.0321 | No |
| 148 | CERS6 |  |  | 865 | -0.362 | 0.0374 | No |
| 149 | SYNE1 |  |  | 877 | -0.365 | 0.0331 | No |
| 150 | PDPN |  |  | 889 | -0.369 | 0.0289 | No |
| 151 | PIGX |  |  | 894 | -0.371 | 0.0308 | No |
| 152 | DPYD |  |  | 909 | -0.377 | 0.0240 | No |
| 153 | FANCA |  |  | 910 | -0.378 | 0.0296 | No |
| 154 | TUBB4B |  |  | 916 | -0.380 | 0.0308 | No |
| 155 | FOXN3 |  |  | 921 | -0.382 | 0.0329 | No |
| 156 | DERA |  |  | 937 | -0.389 | 0.0254 | No |
| 157 | PDGFA |  |  | 949 | -0.395 | 0.0216 | No |
| 158 | TBC1D5 |  |  | 958 | -0.398 | 0.0204 | No |
| 159 | TNFRSF21 |  |  | 964 | -0.399 | 0.0219 | No |
| 160 | MEG3 |  |  | 975 | -0.404 | 0.0190 | No |
| 161 | DCLK2 |  |  | 986 | -0.408 | 0.0162 | No |
| 162 | MORC4 |  |  | 987 | -0.408 | 0.0223 | No |
| 163 | NFATC2IP |  |  | 999 | -0.413 | 0.0186 | No |
| 164 | PCDH9 |  |  | 1019 | -0.427 | 0.0082 | No |
| 165 | TIMELESS |  |  | 1024 | -0.429 | 0.0110 | No |
| 166 | DDB2 |  |  | 1045 | -0.443 | -0.0002 | No |
| 167 | ITPR2 |  |  | 1053 | -0.447 | 0.0003 | No |
| 168 | SERPINB6 |  |  | 1057 | -0.449 | 0.0043 | No |
| 169 | KLHDC8A |  |  | 1059 | -0.451 | 0.0101 | No |
| 170 | ARID5B |  |  | 1095 | -0.467 | -0.0140 | No |
| 171 | UPP1 |  |  | 1101 | -0.469 | -0.0115 | No |
| 172 | UBC |  |  | 1102 | -0.469 | -0.0045 | No |
| 173 | RBBP8 |  |  | 1128 | -0.494 | -0.0193 | No |
| 174 | HSPA4L |  |  | 1131 | -0.497 | -0.0137 | No |
| 175 | TTYH1 |  |  | 1144 | -0.505 | -0.0168 | No |
| 176 | IFI27L2 |  |  | 1145 | -0.506 | -0.0093 | No |
| 177 | FAM111B |  |  | 1151 | -0.514 | -0.0062 | No |
| 178 | TFRC |  |  | 1159 | -0.521 | -0.0046 | No |
| 179 | SEC14L1 |  |  | 1172 | -0.542 | -0.0072 | No |
| 180 | NRCAM |  |  | 1177 | -0.548 | -0.0026 | No |
| 181 | UTRN |  |  | 1178 | -0.551 | 0.0055 | No |
| 182 | SMAD1 |  |  | 1195 | -0.574 | -0.0001 | No |
| 183 | ITGB5 |  |  | 1205 | -0.590 | 0.0007 | No |
| 184 | GRIA4 |  |  | 1238 | -0.669 | -0.0177 | No |
| 185 | F3 |  |  | 1241 | -0.673 | -0.0095 | No |
| 186 | APLP1 |  |  | 1249 | -0.700 | -0.0053 | No |
| 187 | FST |  |  | 1254 | -0.715 | 0.0017 | No |
| 188 | HMGB2 |  |  | 1263 | -0.736 | 0.0055 | No |
| 189 | ZMAT3 |  |  | 1269 | -0.759 | 0.0124 | No |
| 190 | CCNE2 |  |  | 1273 | -0.771 | 0.0212 | No |
| 191 | HIST1H1E |  |  | 1309 | -1.319 | 0.0097 | No |
Table: GSEA details [plain text format]

  

Fig 2: RICHARDS\_NOWAKOWSKI\_SCIENCE\_2017\_U4\_UPREG: Random ES distribution      
 Gene set null distribution of ES for **RICHARDS\_NOWAKOWSKI\_SCIENCE\_2017\_U4\_UPREG**
